# TYPES OF CULTURAL TOURISM PARTICIPATION AMONG KOREAN BABY BOOMERS

**DOI:** 10.1093/geroni/igac059.2469

**Published:** 2022-12-20

**Authors:** Kyungmin Kim, Bon Kim, Hwasung Song, Jeffrey Burr, Gyounghae Han

**Affiliations:** Seoul National University, Seoul, Seoul-t'ukpyolsi, Republic of Korea; Jeju National University, Seoul, Gyeonggi, Republic of Korea; Suwon Research Institute, Suwon-si, Kyonggi-do, Republic of Korea; University of Massachusetts Boston, Boston, Massachusetts, United States; Seoul National University, Seoul, Seoul-t'ukpyolsi, Republic of Korea

## Abstract

Cultural tourism (e.g., attending festivals, visiting museums and heritage sites) is an important part of leisure activity in middle and later life. As they age, midlife adults may anticipate more time for these activities when their family and work demands are less intense. However, the leisure-as-career perspective suggests that preferences, knowledge and skills for leisure and tourism activities develop throughout the life course. This study examines how Korean middle-aged adults participated in cultural tourism activities and whether their willingness to be involved in active leisure in later life differed by their current patterns of cultural tourism activities. We utilized data from the 2014 Korean Baby Boomer Panel Study (N = 4,053; age 51−60). Latent class analyses were applied to five types of activities (i.e., local festivals and events, exhibitions, museums, heritage sites, and international travels), and three distinct patterns were identified. The majority of the Korean baby boomer sample (81%) belonged to “Inactive cultural visitors”—not participating in any type of activities in the past two years, which may reflect persistent time pressures at midlife. “Casual cultural visitors” (11%) mainly visited local festivals and heritage sites. “Serious cultural visitors” (7%) engaged in all types of activities; they appeared to have more time and financial resources. Further, “serious cultural visitors” showed higher willingness to engage in active later life leisure compared to other two patterns, supporting the continuity of leisure/tourism activities. Our findings highlight the importance of prior leisure and tourism behaviors for understanding future leisure activity expectations.

